# Perceived life expectancy and colorectal cancer screening intentions and behaviour: A population-based UK study

**DOI:** 10.1016/j.pmedr.2019.101002

**Published:** 2019-10-21

**Authors:** Rachael H. Dodd, Lindsay C. Kobayashi, Christian von Wagner

**Affiliations:** a The University of Sydney, Faculty of Medicine and Health, School of Public Health, New South Wales 2006, Australia; b Department of Behavioural Science and Health, University College London, Gower Street, London WC1E 6BT, United Kingdom; c Department of Epidemiology, University of Michigan School of Public Health, Ann Arbor, MI 48109, USA

**Keywords:** Colorectal cancer, Perceived life expectancy, Faecal occult blood test, Cancer screening

## Abstract

The relationships between perceived life expectancy (PLE), cancer screening intentions and behaviour are not well understood, despite the importance of remaining life expectancy for the early diagnosis benefits of screening. This study investigates the relationships between PLE and each of: the intention to complete faecal occult blood test (FOBt) screening, ‘ever’ uptake of FOBt screening, and repeat uptake of FOBt screening for colorectal cancer. Data were from the population-representative Attitudes, Behaviour and Cancer UK Survey II (ABACUS II) in England in 2015. Eligible respondents for the present analysis were aged 60–70 years (FOBt eligible age range), who completed the survey question on perceived life expectancy (N = 824). We used logistic regression models to estimate the associations between PLE and the intention to complete screening, ‘ever’ uptake of screening, and repeat uptake of screening, with adjustment for age, gender, occupation-based social grade, marital status, ethnicity, and smoking status. PLE was positively associated with repeated uptake of FOBt (adjusted OR = 2.55; 95% CI: 1.04–6.30 for expecting to live to ≥90 years versus <80 years). Older adults may base decisions to continually participate in cancer screening on their expectations of remaining life expectancy. Future research should investigate the feasibility and acceptability of individualised cancer screening recommendations that take life expectancy into account.

## 1 Introduction

Colorectal cancer (CRC) is the fourth most common cancer and third leading cause of cancer death in the United Kingdom (UK) (Cancer Research UK, 2015, Siegel et al., 2014), but is highly treatable if detected early (Scholefield, 2002, Libby et al., 2012). In the UK, faecal occult blood test (FOBt) screening for the early diagnosis of colorectal cancer is publicly available free of charge to adults aged 60–74 who are invited biennially through postal invitations to complete the screening test at home and mail back the completed test kit. Annual uptake of FOBt screening among the eligible population has consistently been below the national target of 60% and there are wide variations in uptake according to age, sex, ethnicity, and geographic location, ranging from 35 to 61% of the eligible population (von Wagner et al., 2011). The reasons for these low and variable rates of uptake in the population are unknown, and there is interest in optimising uptake rates to ensure that the benefits are maximised and harms minimised among the screening-eligible population.

Importantly, the benefit of CRC screening depends on an individual’s remaining life expectancy. The American College of Physicians recommends that individuals who have less than 10 years of remaining life expectancy should not participate in CRC screening, as the harms they may experience outweigh any benefit from early diagnosis (Levin et al., 2008). Age alone is not sufficient to determine the appropriateness of screening and there is some evidence that older adults may tailor their decision to participate in cancer screening according to their remaining life expectancy. Two existing studies have observed that perceived life expectancy (PLE; also referred to as self-rated life expectancy) (Roberto and Kawachi, 2015) is associated with the uptake of mammography screening for breast cancer in 14 European countries and in Israel (Wuebker, 2012), and the uptake of colorectal cancer screening in England (Kobayashi et al., 2017). Both of these studies indicated that PLE has a strong relationship with actual mortality risk factors, and is concordant with a validated 10-year mortality risk score in England (Kobayashi et al., 2017).

The relationships between PLE and the intention to take up screening in the future, as well as repeated screening uptake behaviour is unknown. Repeated uptake by eligible adults is important to ensure that population-based screening programs remain effective (Lo et al., 2015), and is of particular importance for FOBt screening due to its low sensitivity (Burch et al., 2007). In a population-based study of older men and women living in England, we thus aimed to investigate the relationships between PLE and each of: 1) intention to participate in FOBt screening in the future, 2) ‘ever’ uptake of FOBt screening, and 3) repeated uptake of FOBt screening for colorectal cancer.

## 2 Methods

### 2.1 Design

Data were from the second wave of the Attitudes, Behaviour and Cancer UK Survey (ABACUS II) of 1464 adults in England aged 50–70 in April 2015. The ABACUS II was included as part of the population-representative TNS Research International weekly omnibus survey. The TNS omnibus survey defined sample points using 2001 Census small-area statistics and the Postcode Address File, which are used for random location sampling selection. The sample points were stratified by social grade and Government Office Region. To ensure a sample representative of the general population, quotas were set for age, gender, children at home and working status. The interviews were conducted in respondents’ homes by trained interviewers using face-to-face computer-assisted personal interviews (CAPI). This study was exempt from UCL Research Ethics Committee due to its anonymous and non-sensitive survey methods with non-vulnerable participants.

### 2.2 Study sample

Respondents eligible for the present analysis were within the FOBt-eligible age range (60–70 years 824/1464; 56% of the total sample), who completed the survey question on PLE and the questions on 1) future intentions to complete FOBt screening (n = 816; 99%), 2) ‘ever’ uptake of FOBt screening (asked to those who responded affirmatively to having received an invitation to screening in the past; n = 680; 83%), and 3) repeated uptake of FOBt screening (asked to those who responded affirmatively to having received an invitation to screening in the past; n = 537; 65%). Respondents were excluded if they had a previous diagnosis of bowel cancer (n = 8). The analytic samples included 657, 580 and 415 adults for the models predicting future screening intention, ‘ever’ uptake, and repeat uptake, respectively.

### 2.3 Measures

#### 2.3.1 Sociodemographics

Sociodemographic factors that were thought to be associated with both PLE and each of future screening intention, ‘ever’ uptake, and repeat uptake of screening were included in models as potential confounding variables: age group (60–64; 65–70), gender, marital status (married/living as married; single; widowed/separated/divorced), ethnicity (white; non-white), and social grade according to the occupation of the household primary wage earner (A/B: high or intermediate managerial, administrative, or professional; C1: supervisory, clerical and junior managerial, administrative, or professional; C2: skilled manual workers; D: semi and unskilled workers; E: state pensioners, casual or lowest grade workers, unemployed with state benefits), and current smoking status (never smoker, current smoker, former smoker).

#### 2.3.2 Screening intentions

Respondents were asked, ‘Will you do the stool test the next time you are sent a kit?; the response options were: ‘no, definitely not’; ‘no, probably not’; ‘yes, probably’; ‘yes, definitely’; ‘not sure’). Responses were dichotomised into ‘yes, definitely’ vs all other responses.

#### 2.3.3 Screening uptake: ‘ever’ and repeat uptake

Respondents within the screening-eligible age range (60–70 years) who reported previously receiving an invitation to screening were asked, ‘Have you ever done the stool test?’; the response options were: ‘yes’; ‘no’; ‘don’t know’. ‘Don’t know’ responses were coded as ‘no’, and responses were then dichotomised to reflect ‘never’ vs ‘ever’ screening. To measure repeated uptake of screening, respondents were asked, ‘If you have been sent a test kit more than once, have you done it at least twice?’; response options were: ‘yes, I have done it at least twice’; ‘no, I have only done it once’; ‘don’t know’; ‘not applicable’. Don’t know responses were coded as ‘no’, and the outcome variable was dichotomised to reflect ‘yes’ vs. ‘no’ for repeat screening uptake.

#### 2.3.4 Perceived life expectancy

Respondents were asked, ‘Thinking about your life, what age do you think you will live to?’; response options were: less than 70; 70 to 79; 80 to 89; 90 to 99; 100 or over; don’t know. For analysis, responses were recategorised into ‘less than 80’, ‘80 to 89’ and ‘90 or over’ due to small sample numbers in some categories. Don’t know responses were coded as missing.

#### 2.3.5 Statistical analysis

Characteristics of the sample were described, overall and according to each screening outcome variable. Unadjusted logistic regression models were run initially to estimate the relationships between all covariates and the three screening outcome variables. We then used logistic regression models adjusted for all covariates to estimate the relationships between PLE and each of: i) future intention to complete FOBt screening; ii) ‘ever’ uptake of FOBt screening, iii) repeat uptake of FOBt screening. All a priori-hypothesised potentially confounding variables, described above, were included in the adjusted models.

## 3 Results

The majority of the sample was aged 65–70 (56%), married or living as married (61.1%), or white (95.2%; Table 1). Approximately half were female (51.7%) or never smokers (50.6%). Social grade was evenly distributed across the sample. Overall, 30% of the sample perceived their life expectancy to be < 80 years, 49% perceived that they would live between 80 and 89 years of age, and 22% perceived their life expectancy to be ≥ 90 years of age (Table 1).

**Table 1:** Sample characteristics, overall and according to each outcome variable, ABACUS II, England, 2015.

Characteristic	Total (n = 784)	Screening intentions		‘Ever uptake’		Repeated uptake	
		Yes, definitely N (%)	All other responses N (%)	Yes N (%)	No N (%)	Yes N (%)	No N (%)
*Age group*							
60–64	345 (44.0)	218 (63.2)	127 (36.8)	220 (75.3)	72 (24.7)	133 (74.3)	46 (25.7)
65–70	439 (56.0)	282 (64.2)	157 (35.8)	317 (81.9)	70 (18.1)	269 (88.8)	34 (11.2)
*Gender*							
Male	379 (48.3)	233 (61.5)	146 (38.5)	262 (78.9)	70 (21.1)	201 (82.7)	42 (17.3)
Female	405 (51.7)	267 (65.9)	138 (34.1)	275 (79.3)	72 (20.7)	201 (84.1)	38 (15.9)
*Marital status*							
Married/living as married	479 (61.1)	322 (67.2)	157 (32.8)	338 (79.5)	87 (20.5)	252 (84.0)	48 (16.0)
Divorced, separated, or widowed	222 (28.3)	135 (60.8)	87 (39.2)	150 (81.1)	35 (18.9)	114 (82.0)	25 (18.0)
Single	83 (10.6)	43 (51.8)	40 (48.2)	49 (71.0)	20 (29.0)	36 (83.7)	7 (16.3)
*Ethnicity*							
White	746 (95.2)	484 (64.9)	262 (35.1)	516 (79.0)	137 (21.0)	385 (83.2)	78 (16.8)
Non-white	38 (4.8)	16 (42.1)	22 (57.9)	21 (80.8)	5 (19.2)	17 (89.5)	2 (10.5)
*Occupation-based social grade*							
A/B	164 (20.9)	119 (72.6)	45 (27.4)	131 (88.5)	16 (11.5)	109 (91.6)	10 (8.4)
C1	157 (20.0)	102 (65.0)	55 (35.0)	108 (21.7)	30 (78.3)	72 (75.8)	23 (24.2)
C2	166 (21.2)	106 (63.9)	60 (36.1)	109 (77.3)	31 (22.7)	78 (83.0)	16 (17.0)
D	103 (13.1)	70 (68.0)	33 (32.0)	67 (77.9)	19 (22.1)	64 (97.0)	2 (3.0)
E	194 (24.7)	103 (53.1)	91 (46.9)	122 (73.5)	44 (26.5)	111 (91.0)	11 (9.0)
*Smoking status*							
Never smoker	397 (50.6)	255 (51.0)	142 (50.0)	281 (52.3)	56 (39.4)	210 (52.2)	40 (50.0)
Former smoker	240 (30.6)	167 (33.4)	73 (25.7)	175 (32.6)	45 (31.7)	136 (33.8)	26 (32.5)
Current smoker	147 (18.8)	78 (15.6)	69 (24.3)	81 (15.1)	41 (28.9)	56 (13.9)	14 (17.5)
*Perceived life expectancy*							
<80 years	194 (29.5)	115 (59.3)	79 (40.7)	124 (75.2)	41 (24.8)	87 (77.7)	25 (22.3)
80 to 89 years	322 (49.0)	224 (69.6)	98 (30.4)	239 (81.8)	53 (18.2)	183 (83.9)	35 (16.1)
≥90 years	141 (21.5)	98 (69.5)	43 (30.5)	101 (82.1)	22 (17.9)	77 (90.6)	8 (9.4)

**Table 2:** Perceived life expectancy and screening intentions, ever uptake and screening uptake.

Perceived life expectancy	Total sample (n = 784)	Screening intentions (n = 657)		Ever uptake (n = 580)		Repeated uptake (n = 415)	
		N (%)	OR (95% CI)	N (%)	OR (95% CI)	N (%)	OR (95% CI)
<80 years	194 (29.5)	115 (59.3)	(ref)	124 (75.2)	(ref)	87 (77.7)	(ref.)
80–89 years	322 (49.0)	224 (69.6)	1.27 (0.85–1.89)	239 (81.8)	1.05 (0.63–1.73)	183 (83.9)	1.31 (0.69–2.47)
≥90 years	141 (21.5)	98 (69.5)	1.39 (0.86–2.25)	101 (82.1)	1.21 (0.65–2.23)	77 (90.6)	2.55*(1.04–**6.30)**

* Significant at p < 0.05.

The odds ratios for the associations between PLE ≥90 vs. <80 years and each of screening intentions and ‘ever’ screening uptake were in the positive direction, but with imprecise confidence intervals that crossed the null in fully-adjusted models (screening intention: OR = 1.39, 95% CI: 0.86–2.25; ever screened: OR = 1.21, 95% CI: 0.65–2.23) (Table 2). FOBt screening-eligible adults who perceived their life expectancy to be ≥90 vs. <80 years were more likely to complete FOBt screening at least twice (OR = 2.55, 95% CI: 1.04–6.30; Table 2). There were statistically significant linear trends in a longer PLE being associated with increased odds of future intention to screen (p = 0.058) and repeated FOBt screening (p = 0.006; data not shown).

## 4 Discussion

In this population-based study of older English men and women who were age-eligible for nationally organised CRC screening, we observed consistent trends with previous research that perceived life expectancy is related to cancer screening behavior (Kobayashi et al., 2017). We newly observed that PLE was strongly and positively associated with repeated uptake of screening, with a stronger magnitude than for screening intentions and ever uptake.

As suggested in previous research, some adults may not think life expectancy is important in cancer screening decisions (Schoenborn et al., 2017). People must at first recognise the possibility of contracting a disease before they take personal action (Robb et al., 2004). Previous findings from the UK Flexible Sigmoidoscopy Trial showed that those who perceive their risk of colorectal cancer to be higher than others are more interested in screening (Wardle et al., 2002) and a study in Australia showed those with a high mortality risk were more likely to take up CRC screening (Royce et al., 2014). It is understandable that those who perceive limited life expectancy may perceive limited benefits from screening (Wuebker, 2012); suggesting perception of risk and perception of life expectancy are two different constructs. Both constructs should contribute to decision-making about screening, given the importance of life expectancy in the individual harm-benefit ratio associated with FOBt screening for colorectal cancer.

To ensure that population-based screening programs remain effective, PLE could be used as a measure for targeting those at risk of not attending screening in the future, perhaps based on an inaccurate perception of their remaining life expectancy. Although intention does not always translate into behaviour, known as the ‘intention-behaviour gap’ (Conner et al., 2005), PLE could be one motivating factor that affects intention. As future screening intentions in this study did not distinguish between those who had done the test before or not, past behaviour could have influenced future intentions for screening (Kidwell and Jewell, 2008). Future research could disentangle further the relationship between PLE and its association with screening, along with the recommendations offered by clinicians.

Although we observed point estimates of a strong magnitude between PLE and each of future screening intentions and ‘ever’ screening uptake, the confidence intervals were wide and uninformative. Hence, a limitation is that we had low statistical power to detect the relationships under study; but our results are the first we are aware of on these specific associations and they raise important future questions about the role of PLE in cancer screening uptake. It may be that asking participants about their perceived life expectancy such as in previous research (Kobayashi et al., 2017) may be more appropriate to measure benefits of screening and a stronger effect might be observed. Further, we do not know the accuracy of PLE in this study, but evidence from the English Longitudinal Study of Ageing shows strong correlations between PLE and actual mortality risk among adults aged ≥50 in England (Kobayashi et al., 2017). Our study was cross-sectional, and we could not determine the true causal direction of association between PLE and CRC screening intentions and behaviours. This study provides suggestive findings about PLE and cancer screening intentions and behaviours, which should be investigated prospectively in future research to understand the causal mechanism. A better understanding of the link between perceived life expectancy and perceived benefits of cancer screening is necessary, as we could expect that perceived benefits may mediate this relationship.

Ultimately, this research could inform the targeting of screening to optimise uptake based on life expectancy among adults in the eligible age range. Four areas which could be targeted are: 1) ensuring that perceived life expectancy corresponds with actual life expectancy, 2) increasing knowledge of the age-related benefits of cancer screening, 3) increasing awareness of moderators of life expectancy (including misconceptions) and 4) increasing awareness of mediators of the relationship between life expectancy and uptake (i.e. perceived benefits).

## 5 Conclusion

In conclusion, this study demonstrates that PLE could be influential in the repeated uptake of FOBt screening for age-eligible men and women in England. This is an important finding, as repeated uptake by eligible adults is necessary for the success of population-based screening programs. Future research should determine whether and how to best integrate life expectancy and people’s perceptions of their life expectancy into screening guidelines and informed decision-making processes in the UK. Personalised recommendations from health care professionals that incorporate life expectancy may be valuable for optimising cancer screening uptake rates in the future, as more evidence comes to light about the timeline of benefit from screening, especially among older adults.
